# Case report: Treatment of intraductal papillary mucinous neoplasms located in middle-segment pancreas with end-to-end anastomosis reconstruction after laparoscopic central pancreatectomy surgery through a pigtail-tube-stent placement of the pancreatic duct

**DOI:** 10.3389/fsurg.2022.937682

**Published:** 2022-09-01

**Authors:** Guohua Liu, Xiaoyu Tan, Jiaxing Li, Guohui Zhong, Jingwei Zhai, Mingyi Li

**Affiliations:** Department of Hepatological Surgery, Affiliated Hospital of Guangdong Medical University, Zhanjiang, China

**Keywords:** intraductal papillary mucinous neoplasm (IPMN), laparoscopic, pancreatectomy, anastomosis, case report

## Abstract

Intraductal papillary mucinous neoplasm (IPMN) of the pancreas is one type of pancreatic cystic neoplasm. IPMNs can be classified into three types: main duct-IPMN (MD-IPMN), branch duct-IPMN (BD-IPMN), and mixed type-IPMN (MT-IPMN). It is universally accepted by most surgeons that patients who suffered from MD-IPMN with a high risk of malignant transformation should undergo surgical resection. However, a consensus on the best surgical strategy for MD-IPMN located in the pancreatic neck has still eluded the surgical community worldwide. Recently, one patient suffering from this condition in our Minimally Invasive Pancreas Center underwent a successful surgical procedure. In this case report, we performed a laparoscopic central pancreatectomy for this patient. During this surgical procedure, we used a method of end-to-end anastomosis reconstruction through a pigtail-tube-stent placement of the pancreatic duct. Before the construction of the remnant *pancreas*, the surgical margins of the frozen section should be negative. After surgery, the outcome of this case was satisfactory. No complications such as postoperative hemorrhage, abdominal infection, pancreatitis, delayed gastric emptying, and clinically relevant postoperative pancreatic fistula occurred, which demonstrated that this surgical strategy could achieve a good clinical therapeutic effect for the pancreatic neck MD-IPMN. The result of postoperative routine pathology confirmed the diagnosis of MD-IPMN. The pathological features also showed that there was a high degree of hyperplasia in the local epithelium, which indicated the necessity of surgical treatment.

## Introduction

Intraductal papillary mucinous neoplasm (IPMN) of the pancreas is a mucinous tumor involving the main or branching pancreatic duct. It was first reported in the late 1970s. With improvements in radiographic and endoscopic imaging techniques, IPMN represents approximately 1% of all pancreatic neoplasms (1, 2). IPMNs are divided into three types: main duct-IPMN (MD-IPMN), branch duct-IPMN (BD-IPMN), and mixed type-IPMN (MT-IPMN) based on imaging studies and/or histology (3). Because MD-IPMN has a higher risk (36%–100%) of malignant transformation than the others (4, 5), it is common practice to completely remove the lesion. However, devising appropriate surgical strategies for patients with pancreatic IPMNs is still a challenge (2). For example, there are diverse clinical protocols to completely remove the tumor located in the pancreatic neck and achieve a negative margin, such as pancreaticoduodenectomy (PD), distal pancreatectomy (DP), and central pancreatectomy (CP). According to the Fukuoka guidelines, DP is preferred because it is technically easier to resect additional pancreatic tissue to achieve a negative margin (3). CP has the advantage of preserving the pancreatic parenchyma, thus reducing the occurrence of postoperative endocrine and exocrine insufficiency (6). Besides, the operating procedure of CP is relitavey uncomplicated, compared to PD. Nevertheless, CP has a higher rate of postoperative pancreatic fistula (POPF), which is the main reason for CP not being routinely used in eligible patients (7). Due to improvements in minimally invasive surgery, CP has also been implemented by using the laparoscopic and robotic approaches in recent years (8–10). At present, the reported pancreatic continuous reconstruction after surgery whether in a laparoscopic or robotic operation is done through pancreaticogastrostomy and pancreaticojejunostomy, except in the research reported by Rong et al. (11–13). They first used end-to-end anastomosis after CP through the robotic method. But the mature and safe technique of pancreatic end-to-end anastomosis has not been uniformly used. In this report, we used a method of end-to-end anastomosis reconstruction after laparoscopic central pancreatectomy (LCP) surgery through a pigtail-tube-stent placement of the pancreatic duct and achieved a good therapeutic effect.

## Cases and methods

**Figure 1:**
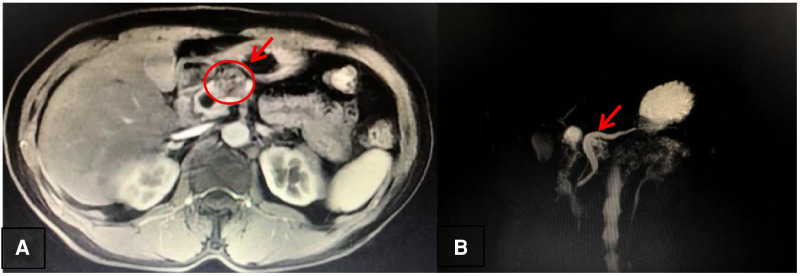
**A**: Magnetic resonance imaging. **B**: Magnetic resonance cholangiopancreatography..

**Figure 2:**
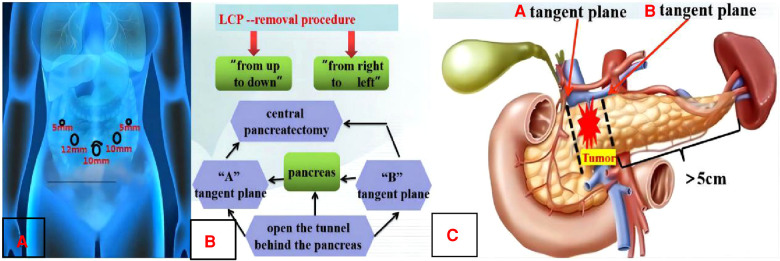
Preoperative design. **A**: Layout of “5 trocar-puncture”. **B**: LCP-removal procedure. **C**: Schematic diagram of CP pancreatic margin.

(1) Case history summary
  (a) A female, 62 years old, was admitted for the treatment of the pancreatic space-occupying lesion found by color ultrasound.
  (b) Main concerns and symptoms of the patient: The patient had no obvious signs and symptoms, and the pancreatic space-occupying lesion was found only by color ultrasound. There had been no previous intervention or treatment for the disease.
  (c) Medical history: Denial of history of diabetes, hypertension, history of infectious diseases, and genetic diseases, denial of history of trauma, surgery, and history of allergies to drugs, food, and other contacts. Family history: Both parents of the patient are alive, and they have one son and another daughter. They are all healthy, without the same disease as the patient, and have no genetic-related disease.
  (d) Physical examination and blood drawing tests (including tumor markers such as CA199 and CEA) showed no obvious abnormalities.
  (e) Imaging examination: One cystic lesion of the pancreatic neck (2.7 cm × 1.8 cm in size) (Figure 1A), with a significantly dilated pancreatic duct (the diameter is greater than or equal to 10 mm) (Figure 1B), was found by magnetic resonance imaging (MRI) and magnetic resonance cholangiopancreatography (MRCP), throwing open the possibility of pancreatic IPMN (not excluding other lesions).
  (f) EUS-FNA: Pathological histology suggested a benign lesion.
(2) Preoperative diagnosis: MD-IPMN of the pancreatic neck
(3) Surgical method: LCP (end-to-end pancreatic anastomosis)
  (a) The reason we chose LCP (end-to-end pancreatic anastomosis) for this case pertained to two aspects: One was that CP can reserve a higher volume of the remanent *pancreas* compared with DP or PD and the end-to-end anastomosis maintained the original continuity of the intestinal tract, which is also in line with the concept of protecting organ function. The other was that laparoscopic surgery may better reflect the concept of minimally invasive medicine.
  (b) Operational procedure:

Artificial pneumoperitoneum was established by using the layout of “5 trocar-puncture” (Figure 2A). Exploration of the organs in the abdominal and pelvic cavity showed that they were not abnormal. Then, the gastrocolic omentum was opened to reveal the pancreas, and the tumor was found in the neck of the pancreas. Therefore, it was decided to carry out middle pancreatic resection.

The first process was surgical resection (Figure 2B): The lower margins of the pancreas were separated at 1 cm from the right side of the tumor. Then, the gaps between the pancreas and the veins including the superior mesenteric vein and the splenic vein should be separated carefully so that the tunnel behind the pancreas could be opened. The middle pancreas and the main pancreatic duct were cut off from the left and right sides of the tumor with an ultrasonic knife when the cutting lines were marked by suturing a traction line on the “A” and “B” tangent plane (Figure 2C). The routinely intraoperative frozen pathology of the removal specimen showed that the pancreatic tumor belonged to the benign lesions and no tumor or tissue infiltration of the high-grade atypia hyperplasia (HGD) was observed at the trans-section margins of the pancreatic parenchyma and duct.

**Figure 3:**
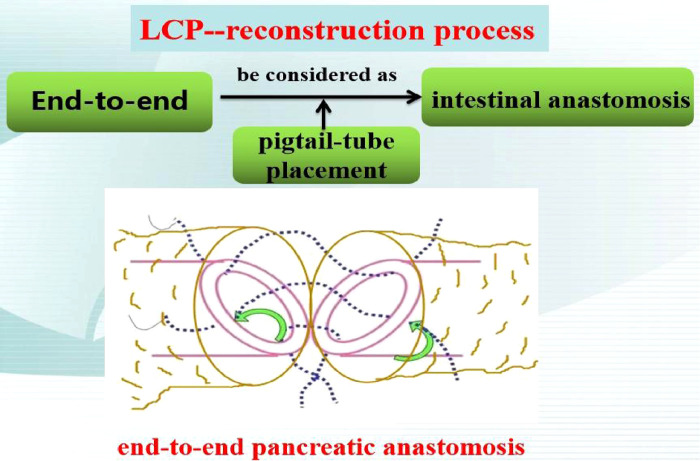
Pancreatic reconstruction process.

**Figure 4:**
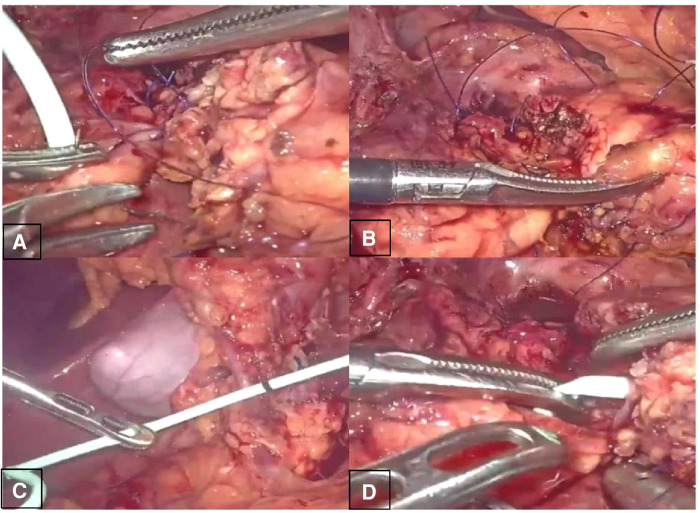
Laparoscopic central pancreatectomy (involving **A**, **B**, **C**, **D** four steps).

**Figure 5:**
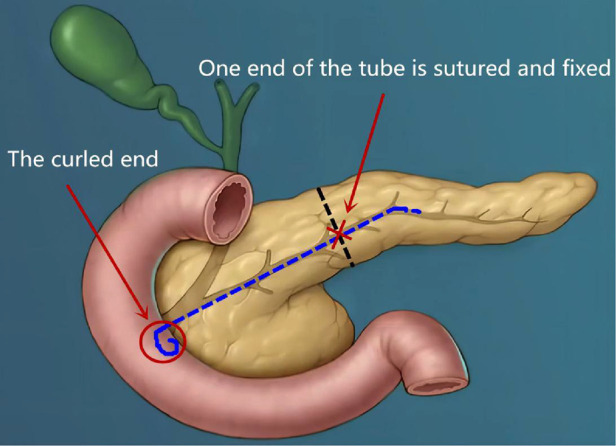
The pigtail position.

The second was the pancreatic reconstruction process (Figure 3): Due to the significantly dilated pancreatic duct and thin pancreatic tissue, in this case, the end-to-end pancreatic anastomosis could be performed analogous to an intestinal anastomosis. The posterior walls of the pancreas and pancreatic duct of the two ends that might be considered the posterior layer of the intestinal anastomosis were continuously sutured with a 4–0 prolene line (Figure 4A). Similarly, the anterior wall of the pancreas and pancreatic duct of the two ends that might also be considered the anterior layer of the intestinal anastomosis was repaired by interrupted 4.0 prolene sutures (Figure 4B). Before the suture of the anterior layer began, we designed a pigtail tube to be placed in the pancreatic duct. One end of this tube was placed in the remanent pancreas and the other end was placed through the duodenal papilla into the duodenum (Figures 4C,D). To avoid stent-tube displacement, one end of the stent tube was sutured and fixed at the end-to-end anastomosis of the pancreas and the curled end would catch the duodenal papilla when the inner core of the stent tube was taken out (Figure 5).

Finally, two drain tubes were placed near the pancreatic wound. Intraoperative bleeding was 20 ml, and the operative time was 140 min.

**Figure 6:**
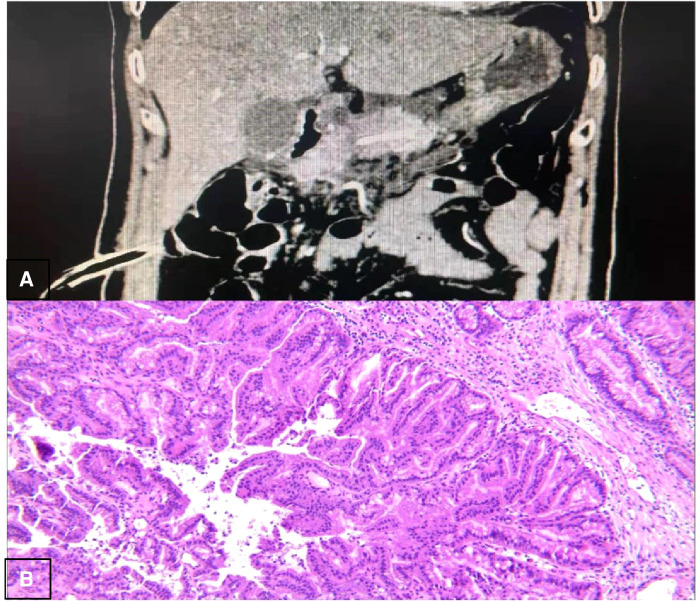
Postoperative outcome: CT and pathological images. **A**: CT images, **B**: pathological images.

**Figure 7:**
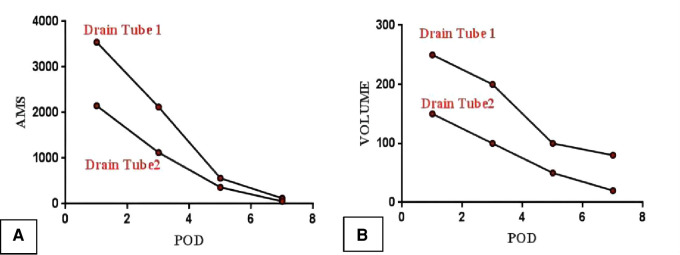
Postoperative outcome: the amylase (**A**) and volume of abdominal drainage tubes (**B**).

(4) Postoperative outcome:
  (a) On the 3rd postoperative day (POD3), the patient had an anal exhaust, so the gastric tube was removed and the fluid diet could be taken out.
  (b) On POD7, the abdominal computed tomography (CT) (Figure 6A) was reviewed and it showed no significant seroperitoneum, so the abdominal drainage tubes were removed.
  (c) On POD12, the patient recovered and was discharged from the hospital.
  (d) On POD1, 3, 5, and 7, the ascitic amylase (Figure 7A) and the volume (Figure 7B) of the two abdominal drainage tubes and the blood results such as C reactive protein (CRP), white blood cell (WBC) count, procalcitonin (PCT), hemoglobin (HGB), and serum amylase (AMS) were tested (Tables 1, 2). The results showed that clinically relevant postoperative pancreatic fistula (cr-POPF) did not occur, although there was a biochemical leak (according to the 2016 ISGPS definition and grading). In addition, other complications such as postoperative hemorrhage (POH), abdominal infection, pancreatitis, and delayed gastric emptying (DGE) did not occur.
  (e) Postoperative pathological images (Figure 6B) showed that the lesion tissue matched the preoperative imaging diagnosis of IPMN. At the same time, HGD was found in the local epithelial tissue.
(5) Postoperative follow-up.

**Table 1:** The amylase and volume of abdominal drainage tubes.

	Drain tube 1		Drain tube 2	
	AMS (U/L)	Volume (ml)	AMS (U/L)	Volume (ml)
POD1	3,545	250	2,150	150
POD3	2,125	200	1,125	100
POD5	560	100	360	50
POD7	120	80	56	20

POD, postoperative day.

**Table 2:** The blood results.

	CRP (mg/L)	WBC (×10^9^/l)	PCT (ng/ml)	Serum AMS (U/dl)	HGB (g/L)
POD1	15.6	16.5	2.5	105	112
POD3	7.1	9.8	0.25	123	110
POD5	2.5	8.6	0.13	43	113
POD7	1.3	7.6	0.18	52	125

POD, postoperative day; PCT, procalcitonin.

The patient was followed up to this day. On 1 and 6 months after the operation, the patient visited the hospital for a physical examination and blood tests for tumor markers and also for a CT scan review. The outcomes displayed that there were no recurrence signs.

## Discussion

Because the pancreatic cystic lesion was accompanied by a significantly dilated pancreatic duct (main pancreatic duct (MPD) diameter ≥ 10 mm), MD-IPMN should be considered for diagnosis in this case. Regardless of the diverse claims on the diameter of the MPD and the size of the cystic lesion, almost all guidelines such as AGA, ACR, and European guidelines suggest that surgery is recommended for MD-IPMN with high-risk stigmata (14). Due to the above reasons, we took surgical treatment measures for this case. After surgical resection, postoperative pathological images showed that the lesion tissue matched the preoperative imaging diagnosis of IPMN and HGD was found in the local epithelial tissue, which confirmed the necessity of the surgical treatment.

In addition, it is still a controversial topic how to choose reasonable methods for the resection range and appropriate surgical approaches for IPMN of the pancreatic neck. For instance, PD, DP, and CP can yield a negative margin for a benign or low-grade malignant tumor of the middle pancreas. Because of the complexity and difficulty of PD, DP and CP have become the mainstream surgical methods. Compared with DP, CP is more difficult to perform and has a higher rate of cr-POPF, which could result in higher mortality (15). Thus, many surgeons may choose DP when confronting this disease. However, CP has the advantage of reserving a large number of pancreatic tissues, which could prevent postoperative endocrine and exocrine insufficiency. Therefore, the question of how to improve the technique of CP has attracted increasing attention from researchers. Most reported studies about anastomosis reconstruction after CP are pancreaticogastrostomy or Roux-en-Y pancreaticojejunostomy (12). Reconstruction of pancreatic end-to-end anastomosis is rarely used for CP, and only some authors have reported this method in an open or robotic operation (11, 16–19), which shows that the anastomotic technique is far from mature. For example, in the study of Rong et al. (19), the group of end-to-end pancreatic anastomosis had a higher incidence of cr-POPF (69.2% vs. 36.4%, *p* = 0.009) and more overall complications than the group of pancreaticojejunostomy. Also, pancreatitis and abdominal infection, which were a result of the relatively serious complications of pancreatic operation, also occurred. Furthermore, this end-to-end pancreatic anastomosis after CP has not yet been used for laparoscopic surgery. This is because it is harder to perform than open or robotic surgery. Therefore, it is very important to improve and simplify this technique to reduce the rate of cr-POPF in laparoscopic surgery.

In this case report, we first applied the approach of end-to-end anastomosis reconstruction to laparoscopic surgery through a pigtail-tube-stent placement of the pancreatic duct. After the surgery, the patient did not develop the symptoms of infection including fever, abdominal distension, and abdominal pain. Besides, the blood results of the patient such as CRP, white blood cell count, and PCT downed to the normal level on POD3, and the serum amylase and hemoglobin remained at normal levels after the operation. The above results showed that POH, DGE, abdominal infection, and pancreatitis did not occur. At the same time, cr-POPF did not occur, although there was a biochemical leak. From the postoperative outcome of this patient, it can be seen that we have achieved a good clinical therapeutic effect by this approach. We think this might be due to the following aspects: (1) As to simplifying the end-to-end anastomotic method after LCP, we treated the walls of the dilated pancreatic duct and the thin pancreatic tissue as the two layers of the anastomosis in this case. The above design made the end-to-end anastomosis easier under laparoscopic conditions and also firmer. (2) As to lowering the incidence rate of cr-POPF after LCP, we drew on our experience from the study of Huscher et al. (20) and devised the following way: One end of a pigtail tube was placed in the remanent pancreatic duct, while the other end was placed through the duodenal papilla into the duodenum. This design might prove more effective for sufficient drainage of pancreatic fluid into the intestinal lumen so as to reduce pancreatic leakage. Moreover, being similar to the lock design, we fixed two ends of the pig tube at the pancreas and the duodenal papilla. In theory, it would be more favorable to avoid cr-POPF than simple stent tube drainage.

## Conclusion

In summary, the method of LCP with end-to-end anastomosis reconstruction after surgery through a pigtail-tube-stent placement of pancreatic duct is feasible, as testified by the fact that the patient in this case report recovered well and cr-POPF did not occur. However, additional evidence will be needed to conclude that the proposed technique effectively reduces cr-POPF and has satisfactory clinical efficacy.

## Data Availability

The original contributions presented in the study are included in the article/Supplementary Material, further inquiries can be directed to the corresponding author/s.
